# Active Control of Stiffness of Tensegrity Plate-like Structures Built with Simplex Modules

**DOI:** 10.3390/ma14247888

**Published:** 2021-12-20

**Authors:** Paulina Obara, Justyna Tomasik

**Affiliations:** Faculty of Civil Engineering and Architecture, Kielce University of Technology, Al. Tysiąclecia Państwa Polskiego 7, 25-314 Kielce, Poland; paula@tu.kielce.pl

**Keywords:** tensegrity plate-like structures, Simplex module, self-stress state, infinitesimal mechanism, qualitative analysis, non-linear quantitative analysis

## Abstract

The aim of this study is to prove that it is possible to control the static behavior of tensegrity plate-like structures. This possibility is very important, particularly in the case of deployable structures. Here, we analyze the impact the support conditions of the structure have on the existence of specific characteristics, such as self-stress states and infinitesimal mechanisms, and, consequently, on the active control. Plates built with Simplex modules are considered. Firstly, the presence of the specific characteristics is examined, and a classification is carried out. Next, the influence of the level of self-stress state on the behavior of structures is analyzed. A geometrically non-linear model, implemented in an original program, written in the Mathematica environment, is used. The results confirm the feasibility of the active control of stiffness of tensegrity plate-like structures characterized by the presence of infinitesimal mechanisms. In the case when mechanisms do not exist, structures are insensitive to the initial prestress level. It is possible to control the occurrence of mechanisms by changing the support conditions of the structure. Based on the obtained results, tensegrity is very promising structural concept, applicable in many areas, when conventional solutions are insufficient.

## 1. Introduction

Tensegrity is a relatively new structural concept, its origins dating to the 1960s. At first, tensegrities were found in arts, to a lesser degree in architecture and, consequently, in civil engineering. Currently, due to their universality, the use of tensegrity systems is gradually increasing in many fields, from micro- to macroscale. Tensegrity models can be used to describe the mechanical behavior of living cells [1,2], in biomedicine (biotensegrity) [3,4], in mechanical engineering [5,6], or as a new material called metamaterial [7,8,9,10]. An application of the tensegrity concept in civil engineering is still quite innovative and the interest of architects and engineers in the practical application of this solution is growing. In the past few years, numerous projects and implementations of the tensegrity idea have been created. The attractiveness of tensegrity structures arises from designers striving for originality and innovation. Tensegrities are interesting despite being made from the simplest possible elements. These spatial, light-weight structures are composed of compressed elements (struts or rods), separated from each other, floating inside the continuous net of tensed elements (cables). The components of the tensegrity system are assembled in a self-balancing way, meaning that there is an equilibrium stress state among struts and cables under zero external loads. This configuration of internal forces is called a “self-stress state”. Self-stress states stabilize infinitesimal mechanisms, the occurrence of which is another immanent feature of tensegrity structures. It should be noted that in the literature, structures without mechanisms are also described as tensegrities. The identification of mechanisms and the correct classification of tensegrity structures is very important due to different behavior of the structure under external actions.

The most dominant subject in the literature, starting from the beginning of the idea of tensegrity to the present day, is the search of the geometrical configuration (the form-finding) of tensegrity structures, as seen in a sample of papers from the past two years [11,12,13,14,15]. In turn, the most interesting subject is using tensegrity as deployable structures with active control. Tensegrity systems offer many advantages over conventional structural systems—they have higher load-bearing capacity than conventional structures with the same mass, occupy less space and it is easy to change their geometrical configuration due to the mechanisms. The possibility of the control of the behavior of the structure throughout the adjustment of the level of self-stress state forces of structure is very promising. Over the years, many studies in this area have been carried out. In [16,17], the use of the pentagonal tensegrity-ring module in an active deployable footbridge is explored. The circuit-pattern based module allows for reducing the number of actuated cables. The authors adopted a mechanism-based deployment strategy, meaning that they change cable lengths to introduce finite mechanisms and then find corresponding equilibrium configuration. A similar approach, exploiting the existence of the mechanisms, is presented in [18]. In Reference [19], the proposed application of a foldable, curved tensegrity double-layered grid also exploited the presence of infinitesimal mechanisms. In Reference [20], a strategy for the deployment of tensegrity systems is presented. According to the presented idea, every deployment configuration is close to the stable equilibrium state, so the structure can be easily transformed from one state to another. In Reference [21], clustered actuation of tensegrity structures is presented. In that approach, cables run through frictionless pulleys or loops at the end of struts. In References [22,23], the behavior of an asymmetrical, five-module structure built with telescopic struts is explored. The authors developed a quasi-static strategy based on a stochastic search algorithm combined with dynamic relaxation for controlling the geometry of the structure. In References [7,24], the application of tensegrity systems as smart structures is explored. The authors presented how the active control of prestressing forces in single and multi-module structures enables adapting the structure to the actual environmental conditions.

One of the numerous applications of the tensegrity principle in civil engineering is in double-layer grids. Generally, the elements of a double-layer grid are organized into two parallel planes, which are connected by vertical and diagonal elements. In the horizontal projection, the elements are arranged in a regular pattern. Double-layer grids are similar to plates and therefore, they are called tensegrity plate-like structures or tensegrity plates. These structures are built from the basic tensegrity modules such as Simplex [25,26,27,28,29,30,31,32]. For example, in Reference [28], the first experimental model of the tensegrity plate built with thirty-three modified Simplex modules was created. In Reference [29], the properties of tensegrity structures built from the same module, but connected in different ways, were compared. In Reference [30], a method of obtaining tensegrity panels based on the geometry of traditional double-layer trusses was proposed, while in Reference [31], constructions with single and double curvatures were studied.

This paper concerns the application of tensegrity plate-like structures as deployable structures with active control of stiffness. The review of the literature showed that relatively few works investigate the control of stiffness in tensegrity structures by the modification of the self-stress state [33,34,35,36,37,38,39,40,41,42]. In those papers, the static or dynamic parameters in the function of the prestress forces were designated. It was proved that the control of stiffness is possible only for structures with infinitesimal mechanisms. The guiding question here is as follows: is it possible to control the occurrence of mechanisms by changing the support conditions of the structure? Taking into account deployable structures, for which the support conditions could be changed, this is a very legitimate problem. The next interesting question can be stated: does the behavior of plates with mechanisms with the same geometry alter with the change in support conditions?

To answer these questions, we carried out a parametric analysis and analyzed the influence of the initial prestress on the static behavior of tensegrity plate-like structures. The plates built with a Simplex module were considered. The “normal” module and a modified one were taken into account. Many options for structures, including different possibilities of support, were studied. The impact of the support conditions of the structure on the existence of mechanisms and consequently on the active control was analyzed. The complete analysis of tensegrity structures contains qualitative and quantitative assessments. In the first stage, the characteristic features of tensegrity structures were identified. On this basis, the plate-like structures were classified into four groups. This classification is important for the quantitative analysis because the structures behave differently under external actions. The second stage focused on the behavior of tensegrity systems under external loads. In particular, the influence of initial prestress level on the displacements, effort and stiffness of the structures was analyzed. To the best of our knowledge, there is no such full consideration in the current literature.

Due to their specific structure, tensegrity plates can be analyzed using a discrete model or continuum one. In this paper, a discrete model using the finite elements method is applied. In the future, for the comparison, the continuum model will be used.

## 2. Materials and Methods

**Figure 1:**
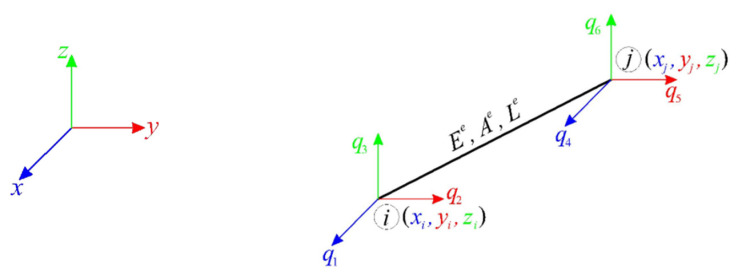
Space truss finite element e.

Tensegrity plate-like structures are spatial lattice systems in a self-stress state. The specificity of tensegrity lies in the fact that the self-stress states stabilize the existing (if any) infinitesimal mechanisms. In order to analyze these structures, the finite elements method is used [43,44]. In a global coordinate system (x, y, z), a finite element e (Figure 1) is described by Young’s modulus $E^{e}$, cross-sectional area $A^{e}$, length $L^{e}$ and compatibility matrix $B^{e}\left(\in {\mathbb{R}}^{1\times 6}\right)$ [45]:

(1) $$B^{e}=\left[\begin{array}{cccccc} -c_{x} & -c_{y} & -c_{z} & c_{x} & c_{y} & c_{z} \end{array}\right],$$

where: $c_{x}=\frac{x_{j}-x_{i}}{L^{e}},{\;c}_{y}=\frac{y_{j}-y_{i}}{L^{e}},{\;c}_{z}=\frac{z_{j}-z_{i}}{L^{e}}.$

.

An analyzed n-element space truss (e=1, 2,…, n) with m-degrees of freedom is described by a displacement vector $q\left(\in {\mathbb{R}}^{m\times 1}\right)$, an extension vector $\Delta \left(\in {\mathbb{R}}^{n\times 1}\right)$ and a vector of longitudinal forces $\;S\left(\in {\mathbb{R}}^{n\times 1}\right)$:

(2) $$q=[\begin{array}{cccc} q_{1} & q_{2} & \ldots & q_{m} \end{array}{]}^{T};\;\;\Delta =[\begin{array}{cccc} \Delta_{1} & \Delta_{2} & \ldots & \Delta_{m} \end{array}{]}^{T};\;\;S={\left[\begin{array}{cccc} S_{1} & S_{2} & \ldots & S_{n} \end{array}\right]}^{T}.$$

The relations between parameters in (2) are described by three equations, specifically, compatibility, material properties and equilibrium ones, with boundary conditions included:

(3) $$\;\;\Delta =Bq;\;\;S=E\Delta ;\;B^{T}S=P,$$

where $B\left(\in {\mathbb{R}}^{n\times m}\right)$ is an expansion matrix, **P**$\left(\in {\mathbb{R}}^{n\times 1}\right)$ is an external load vector and $E\left(\in {\mathbb{R}}^{n\times n}\right)$ is an elasticity matrix $E\left(\in {\mathbb{R}}^{n\times n}\right)$:

(4) $$E=diag\left[\begin{array}{cccc} \frac{E^{1}A^{1}}{L^{1}} & \frac{E^{2}A^{2}}{L^{2}} & \ldots & \frac{E^{n}A^{n}}{L^{n}} \end{array}\right].$$

The complete analysis of tensegrity structures is a two-stage process. The first stage is a qualitative analysis, and the second is a quantitative analysis.

### 2.1. Qualitative Analysis

The qualitative analysis is the first step to understand unique properties of tensegrity structures. This assessment is required to determine the immanent features such as infinitesimal mechanisms and self-equilibrium systems of longitudinal forces (self-stress states) which stabilize mechanisms [45,46]. It is possible that self-stress states also occur in geometrically invariable structures.

To identify characteristic features of tensegrities, the spectral analysis of the truss matrices is used. The equilibrium Equation (3)_3_ can be presented in the form of stresses or in the form of displacements:

(5) $$\;DS=BP;\;\;\;\;\;\;\;\;K_{L}q=P,$$

where $D=BB^{T}$ is the compatibility matrix and $K_{L}=B^{T}EB$ is the linear stiffness matrix. The spectral analysis of the matrix $D\left(\in {\mathbb{R}}^{n\times n}\right)$ leads to identifying self-stress states, while that of the matrix $K_{L}\left(\in {\mathbb{R}}^{m\times m}\right)$ identifies mechanisms:

(6) $$\left(D-\mu I\right)y=0;\;\;\;\;\;\;\;\;\;\;\;\;\left(K_{L}-\lambda I\right)x=0.$$

Solutions of Equation (6) can be expressed as vectors:

(7) $$H=\left\{\begin{array}{cccc} \mu_{1} & \mu_{2} & \ldots & \mu_{n} \end{array}\right\};\;\;\;\;\;L=\left\{\begin{array}{cccc} \lambda_{1} & \lambda_{2} & \ldots & \lambda_{m} \end{array}\right\}.$$

The self-stress state can be considered as an eigenvector $y_{i}=S\left(\mu_{i}=0\right)$ related to zero eigenvalue of the matrix (7)_1_, if any, whereas the mechanism can be understood as an eigenvector $x_{i}=q\left(\lambda_{i}=0\right)$ related to zero eigenvalue of the matrix (7)_2_, if any.

If the self-stress state is defined $S\left(\mu_{i}=0\right)$, the geometric stiffness matrix $K_{G}\left(S\right)\left(\in {\mathbb{R}}^{m\times m}\right)$ is built. The full solution of the eigen problem is provided by the spectral analysis of the stiffness matrix with regard to the effect of self-equilibrated forces:

(8) $$\left(K_{L}+K_{G}\left(S\right)-\sigma I\right)z=0.$$

If the eigenvalues of (8):

(9) $$O=\left\{\begin{array}{cccc} \sigma_{1} & \sigma_{2} & \ldots & \sigma_{n} \end{array}\right\}$$

are positive numbers, the mechanism is infinitesimal and the structure is stable. Zero eigenvalues are related to finite mechanisms, whereas negative eigenvalues are responsible for the instability of the structure.

The self-stress states (*S*) and infinitesimal mechanisms (*M*) are the two most important features of tensegrity structures. There are four more characteristic features: the structure is a truss (*T*), compressed elements (struts) form a discontinuous set so extremities do not touch each other (*D*), tensile elements are cables and have no rigidity in compression (*C*) and the set of struts is contained within the continuous net of tensile elements (*I*). The two first depend on the geometry, whereas the last two features are indirectly recognized by the identified self-stress state. The classification of tensegrity structures, based on the presence of those features, is introduced [45]. The plates can be classified into one of three groups, i.e., ideal tensegrity, and structures with tensegrity features of class 1 or class 2. “Pure” tensegrity, described in [45], does not occur in analyzed cases. This classification is important due to the different behavior of the structure under external actions. This is also especially crucial in the case of deployable structures, for which the possibility to control their behavior is expected.

### 2.2. Quantitative Analysis

The quantitative assessment is the second step of analysis of tensegrity structures. In this step, the impact of the level of self-stress state (initial prestress) on the behavior of tensegrity structures under static load is analyzed. To evaluate this behavior, a geometrically non-linear model is used [42,47,48]. As a basis for formulating the tensegrity lattice equations, the nonlinear theory of elasticity in Total Lagrangian (TL), Lagrange’s stationary description) approach is adopted.

The incremental static equilibrium equation for structures takes the form:

(10) $$K_{T}\left(q\right)\Delta q=\Delta P+R,$$

where $R\left(\in {\mathbb{R}}^{m\times 1}\right)$ is the residual force vector and $K_{T}\left(q\right)\left(\in {\mathbb{R}}^{m\times m}\right)$ is the tangent stiffness matrix of structure presented as:

(11) $$K_{T}\left(q\right)=K_{L}+K_{G}\left(S+N\right)+K_{NL}\left(q\right);\;\;\;K_{NL}\left(q\right)=K_{u1}+K_{u2}.$$

The explicit matrices forms, such as the geometric stiffness matrix $K_{G}\left(S+N\right)$ and the non-linear displacement stiffness matrix $K_{NL}\left(q\right)$**,** can be found, for example, in [42].

To solve the system of the non-linear Equation (10), an original program, written in the Mathematica environment, is used. The Newton–Raphson method is implemented. The program makes it possible to freely define the geometry of the structure, material parameters and loads, and then identify the self-stress states and track the behavior of selected static and geometric parameters in the function of this state.

In this paper, we perform a parametric analysis, which leads to the determination of the impact of initial prestress level S on the behavior of the structure. The analysis contains:

- Determination of the minimum initial prestress level $S_{min}$, corresponding to the lowest level of prestress that ensures an appropriate identification of the type of element (cables or struts);
- Determination of the maximum initial prestress level $S_{max}$, which depends on the load-bearing capacity of the most stressed elements;
- Assessment of the influence of initial prestress level on the displacements q; and
- Assessment of the influence of the initial prestress level on the effort of the structure:
  (12) $$W_{\max}=N_{\max}/N_{Rd},$$
  where $N_{\max}$ is the maximum normal force and $N_{Rd}$ is the load-bearing capacity. Normal forces N are determined as a function of the initial prestress forces S:
  (13) $$N=y_{i}S,$$
  where $y_{i}$ is the normalised vector of the self-stress state determined in the qualitative analysis.

Finally, our parametric analysis contains an assessment of the influence of the initial prestress on the rigidity the structure, determined by the global stiffness parameter (GSP) [42]:

(14) $$GSP=\frac{\;{\left[q\left(S_{min}\right)\right]}^{T}K_{S}\left(S_{min}\right)q\left(S_{min}\right)}{{\left[q\left(S_{i}\right)\right]}^{T}K_{S}\left(S_{i}\right)q\left(S_{i}\right)},$$

where $K_{S}\left(S_{min}\right)$ and $q\left(S_{min}\right)$ are a secant stiffness matrix and a design displacement vector with a minimum initial prestress level, and $K_{S}\left(S_{i}\right)$ and $q\left(S_{i}\right)$ are at i–th prestress level.

## 3. Results

In this paper, the influence of the support conditions on the behavior of plate-like structures is explored. The plates built with the simplest tensegrity modules, i.e., Simplex modules, are considered. A “normal” Simplex module and a modified one are taken into account. The modified module differs from the traditional one in how the top plane of the module is composed. In the modified module, the top plane is inscribed onto the bottom one, making the module easier to connect with the others.

As the first step, the single “normal” Simplex (S1) and modified Simplex (MS1) modules are analyzed. Next, the tensegrity plate-like structures built with single modules are considered. In plates made of the S1 module, the units are connected node-to-node, while in plates built with the MS1 module, top surfaces of the units are connected node-to-node, while the bottom surfaces are connected element-to-element. Six- and twenty-four-module structures are taken into account. Firstly, the qualitative analysis of the structures is performed, and then, the quantitative analysis is conducted.

### 3.1. Qualitative Analysis

The qualitative analysis leads us to identify characteristic features of tensegrity structures. Because geometrical and mechanical characteristics do not affect the unique properties of tensegrity, all constants were assumed as unitary; hence, the elasticity matrix is a unit matrix E=I. The results of the quantitative analysis of the single modules are presented explicitly, according to Section 2.1. The values on the self-stress forces are normalized in such a way that the maximum compressed force in struts is equal to −1. For tensegrity plate-like structures, only the number of identified mechanisms and self-stress states is shown. In the figures presented in the next sections, struts are always marked in black, top cables in green, middle cables in blue and bottom cables in red.

#### 3.1.1. Single Simplex Module

**Figure 2:**
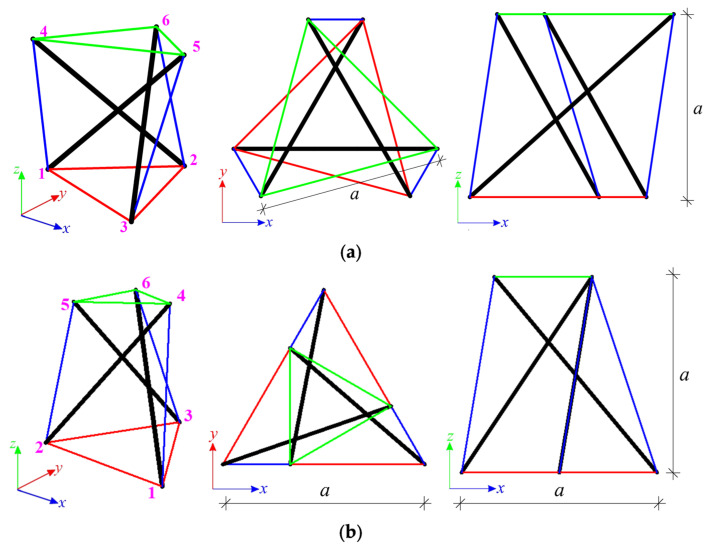
Single Simplex modules: (**a**) S1, (**b**) MS1.

**Figure 3:**
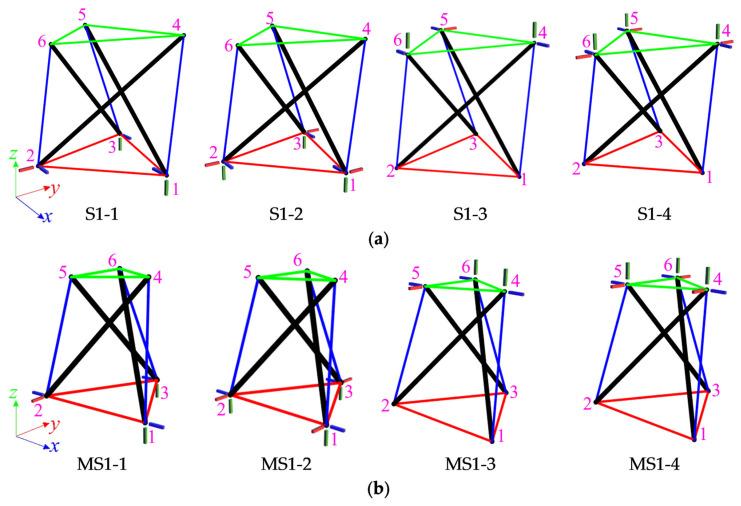
Scheme of support: (**a**) S1, (**b**) MS1.

The first considered structures are the Simplex modules, i.e., S1 (Figure 2a) and SM1 (Figure 2b). Both types of module consist of twelve elements (n = 12), i.e., three struts and nine cables, and six nodes (w = 6). The numeration of elements for the single module is provided in Table 1. For each module, four static schemes are taken into account (Figure 3).

Preliminarily, the support in the bottom nodes is considered and models with six bonds (S1-1, MS1-1) and with nine bonds (S1-2, MS1-2) are analyzed. In the first case, the number of elements and the number of degrees of freedom are equal (n *=* m *=* 12); thus, the matrices (6) are equal ($D\left(\in {\mathbb{R}}^{12\times 12}\right)$**,** $K_{L}\left(\in {\mathbb{R}}^{12\times 12}\right)$). The eigenvalues (7) are the same and do not greatly differ for each model:

 S1-1: H=L={3.75 2.42 2.26 1.65 1.45 1.24 1.08 0.75 0.12 0.10 0.04 0.00},

 MS1-1: H=L={3.57 2.71 2.21 2.02 1.73 1.2 1.06 1.01 0.22 0.04 0.04 0.00}.

**Figure 4:**
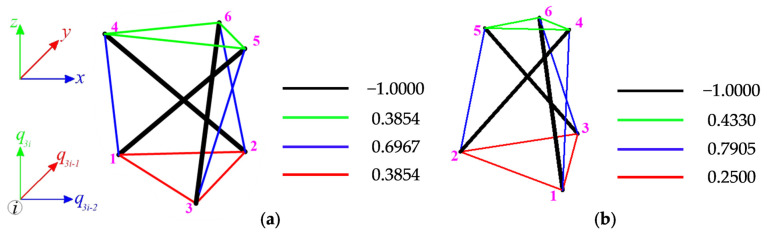
Normalized self-stress state of model: (**a**) S1-1, (**b**) MS1-1.

**Figure 5:**
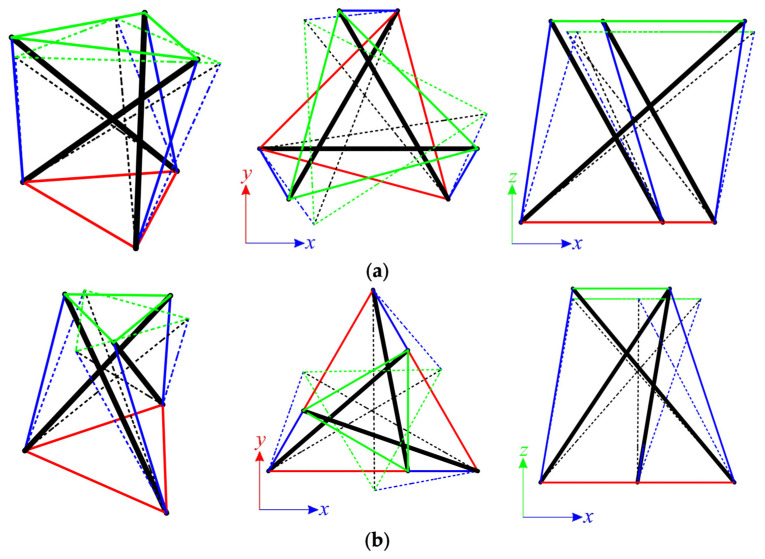
Infinitesimal mechanism of model: (**a**) S1-1, (**b**) MS1-1.

One zero eigenvalue in the above matrices means one self-stress state $y_{12}=S\left(\mu_{12}=0\right)\;$(Figure 4) and one mechanism $x_{12}=q\left(\lambda_{12}=0\right)$ (Figure 5) is identified:

- For the S1-1 model:

$$y_{12}=\left[-1.0\;-1.0\;-1.0\;0.39\;0.39\;0.39\;0.39\;0.39\;0.39\;0.70\;0.70\;0.70\;\right],$$

$$x_{12}=\left[0.0\;0.0\;0.0\;0.39\;-0.39\;0.16\;0.14\;0.54\;-0.16\;-0.54\;-0.14\;-0.16\right],\;$$

- For the MS1-1 model:

$$y_{12}=\left[-1.0\;-1.0\;-1.0\;0.43\;0.43\;0.43\;0.25\;0.25\;0.25\;0.79\;0.79\;0.79\right],$$

$$x_{12}=\left[0.0\;0.0\;0.0\;0.0\;0.55\;-0.16\;0.48\;-0.28\;-0.16\;-0.48\;-0.28\;-0.16\right].$$

The infinitesimal mechanisms of S1 and SM1 modules are quite similar to each other. The top nodes of both structures rotate and lower. The positive eigenvalues of (9):

S1-1: O={4.60 3.75 3.22 2.79 2.28 2.18 1.60 1.18 0.92 0.16 0.13 0.04},

MS1-1: O={7.91 5.47 5.15 3.66 3.57 3.46 2.14 1.75 1.04 0.58 0.14 0.08},

confirm the stability of both modules. The identified mechanism $x_{12}$ is infinitesimal and it is realized by the displacements of the top nodes (Figure 5). The qualitative analysis confirms the existence of the self-stress state and the mechanism, so the models S1-1 and MS1-1 can be qualified as an ideal tensegrity.

For models S1-2 and MS-2, the number of elements and the number of degrees of freedom are not equal (n *=* 9, m *=* 12), and the eigenvalues of the matrices $D\left(\in {\mathbb{R}}^{12\times 12}\right)$ are presented as:

S1-2: H={3.67 2.23 2.23 1.32 1.14 1.14 0.11 0.11 0.0 0.0 0.0 0.0},

MS1-2: H={3.47 2.06 2.06 1.53 1.29 1.29 0.14 0.0 0.0 0.0 0.0}.

while the eigenvalues of the matrix $K_{L}\left(\in {\mathbb{R}}^{9\times 9}\right)$ as:

 S1-2: L={3.67 2.23 2.23 1.32 1.14 1.14 0.11 0.11 0.0},

MS1-2: L={3.47 2.06 2.06 1.53 1.29 1.29 0.14 0.0}. 

In this case, four self-stress states $y_{9-12}=S\left(\mu_{9-12}=0\right)\;$ and one mechanism $x_{9}=q\left(\lambda_{9}=0\right)$ are identified:

- For the S1-2 model:

$$\begin{array}{l} y_{9}=\left[-1.0\;-1.0\;-1.0\;0.39\;0.39\;0.39\;0.0\;0.0\;0.0\;0.70\;0.70\;0.70\right],\\ y_{10}=\left[-1.0\;-1.0\;-1.0\;0.39\;0.39\;0.39\;-49.8\;314.1\;7904.6\;0.70\;0.70\;0.70\right],\\ y_{11}=\left[0.0\;0.0\;0.0\;0.0\;0.0\;0.0\;-0.56\;1.0\;0.04\;0.0\;0.0\;0.0\right],\\ y_{12}=\left[0.0\;0.0\;0.0\;0.0\;0.0\;0.0\;1.0\;0.56\;0.03\;0.0\;0.0\;0.0\right], \end{array}$$

$$x_{9}=\left[0.39\;-0.39\;-0.16\;0.14\;0.54\;-0.16\;-0.54\;-0.14\;-0.16\right],\;$$

- For the MS1-2 model:

$$\begin{array}{l} y_{9}=\left[-1.0\;-1.0\;-1.0\;0.43\;0.43\;0.43\;1.69\;-8.47\;3.15\;0.79\;0.79\;0.79\right],\\ y_{10}=\left[-1.0\;-1.0\;-1.0\;0.43\;0.43\;0.43\;-0.11\;0.54\;-0.20\;0.79\;0.79\;0.79\;\right],\\ y_{11}=\left[0.0\;0.0\;0.0\;0.0\;0.0\;0.0\;1.0\;0.28\;0.23\;0.0\;0.0\;0.0\right],\\ y_{12}=\left[0.0\;0.0\;0.0\;0.0\;0.0\;0.0\;-0.31\;0.30\;1.0\;0.0\;0.0\;0.0\right], \end{array}$$

$$x_{9}=\left[0.0\;-0.55\;-0.16\;-0.48\;0.28\;-0.16\;0.48\;0.28\;-0.16\right].\;$$

The full solution to the eigen problem is provided by the spectral analysis of the stiffness matrix $K_{T1}$, taking into account all four identified self-stress states:

- For the S1-2 model:

$$\begin{array}{c} O_{9}=\left\{4.56\;3.09\;3.09\;2.19\;2.19\;1.51\;0.86\;0.16\;0.16\right\},\\ O_{10}=\left\{4.56\;3.09\;3.09\;2.19\;2.19\;1.51\;0.86\;0.16\;0.16\right\},\\ O_{11}=\left\{3.42\;1.95\;1.95\;1.20\;1.07\;1.07\;0.09\;0.09\;0.00\right\},\\ O_{12}=\left\{3.42\;1.95\;1.95\;1.20\;1.07\;1.07\;0.09\;0.09\;0.00\right\}. \end{array}$$

- For the MS1-2 model:

$$\begin{array}{c} O_{9}=\left\{7.77\;5.11\;5.11\;3.50\;3.50\;2.46\;1.16\;0.18\;0.18\right\},\\ O_{10}=\left\{7.77\;5.11\;5.11\;3.50\;3.50\;2.46\;1.16\;0.18\;0.18\right\},\\ O_{11}=\left\{5.52\;\;2.87\;\;2.7\;\;1.37\;\;1.29\;\;1.29\;\;0.12\;\;0.12\;\;0.00\right\},\\ O_{12}=\left\{5.52\;\;2.87\;\;2.7\;\;1.37\;\;1.29\;\;1.29\;\;0.12\;\;0.12\;\;0.00\right\}. \end{array}$$

For both models, two out of four identified self-stress states ($y_{11},\;y_{12})$ do not stabilize the structures. Additionally, only one self-stress states ($y_{9})$ provides an appropriate identification of the type of elements. In this case, superpositions of all the self-stress states are required. The superposition leads to prestress forces obtained for models S1-1 and MS1-1, and then all eigenvalues of $K_{T1}$ are positive:

$$S1\text{-}2:\;O_{\sup}=\left\{4.55\;3.09\;3.09\;2.19\;2.19\;1.52\;0.86\;0.16\;0.16\right\},$$

$$\mathrm{MS}1\text{-}2:\;O_{\sup}=\left\{5.69\;4.29\;4.29\;3.49\;3.49\;2.52\;1.28\;0.22\;0.22\right\}.$$

Thus, the models S1-2 and MS1-2 are structures with tensegrity features of class 1.

**Figure 6:**
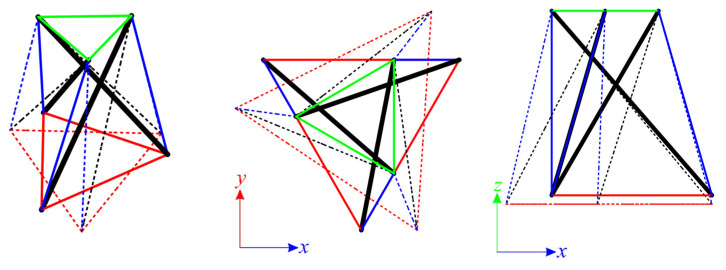
Infinitesimal mechanism of MS1-3 and MS1-4 models.

Next, the support in the top nodes is considered and models with six bonds (S1-3, MS1-3) and with nine bonds (S1-4, MS1-4) are analyzed. The analysis shows that it does not matter whether bottom or top nodes are supported. It was obvious in the case of “normal” modules (S1-3, S1-4), but not for modified modules (MS1-3, MS1-4). The only difference is in the fact that the mechanism is different, and it is realized by the displacements of the bottom nodes (Figure 6). The summarized results of the qualitative analysis of all single modules are contained in Table 2.

#### 3.1.2. Six-Module Simplex Plate-like Structures

**Figure 7:**
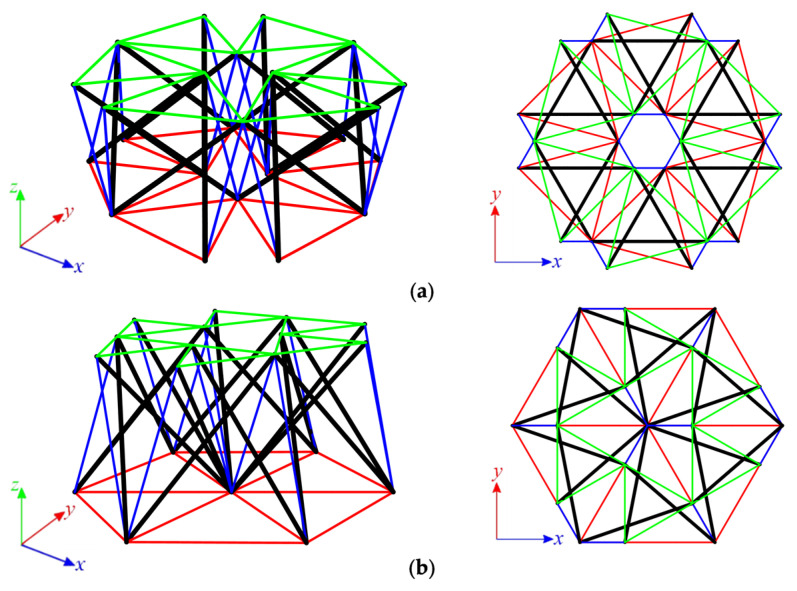
Six-module Simplex plate: (**a**) S6, (**b**) MS6.

**Figure 8:**
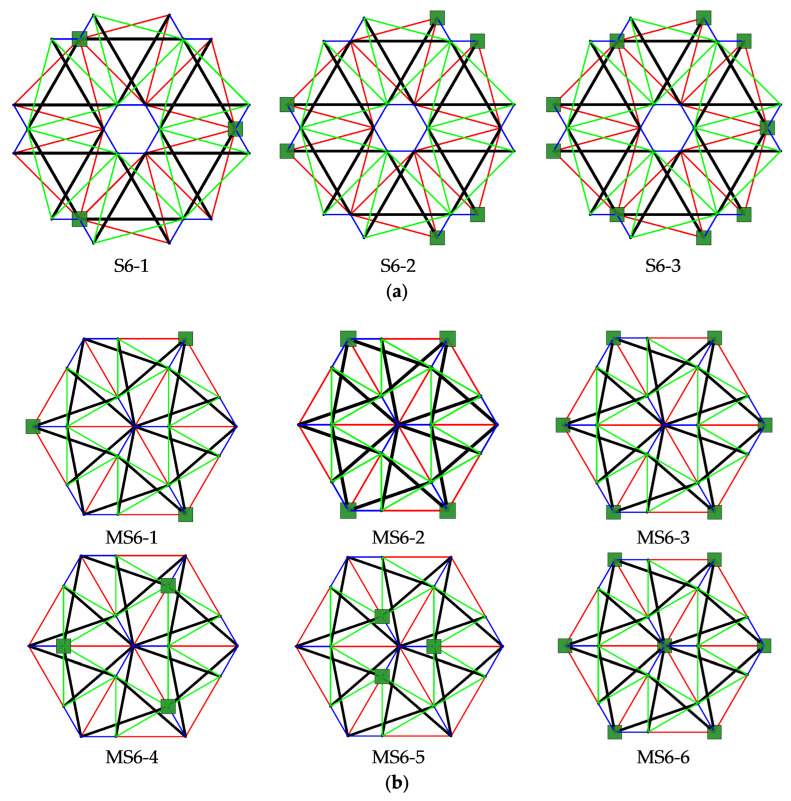
Scheme of support: (**a**) S6, (**b**) MS6.

The structures built with six Simplex modules are considered next. Two simple modules, e.g., S1 and MS1, are used to build the plates. In the first case (Figure 7a), structures consist of 72 elements (n *=* 72) and 24 nodes (w *=* 24), whereas in the second (Figure 7b), n *=* 60 and w *=* 19. Nine simply supported plates, with a different number of supported nodes, are taken into account (Figure 8).

Each analyzed six-module plate features the presence of the self-stress states. The number of self-stress states differs regarding the support conditions. Mechanisms are identified for models S6-1, S6-2, and MS6-1 to MS6-4. For each model, one mechanism is present. As identified self-stress states do not identify the type of elements correctly, a superimposed self-stress state from the single module is taken into consideration. For all models characterized by the existence of mechanisms, the eigenvalues of the tangential stiffness matrix $K_{T1}$ are positive, so structures are considered stable. Models S6-1, S6-2, and MS6-1 to MS6-4 are qualified as structures with tensegrity features of class 1 because of the presence of self-stress states. The rest of the models, not having the mechanisms, are classified as structures with tensegrity features of class 2. The results of the qualitative analysis are summarized in Table 3.

#### 3.1.3. Twenty-Four-Module Simplex Plate-Like Structure

**Figure 9:**
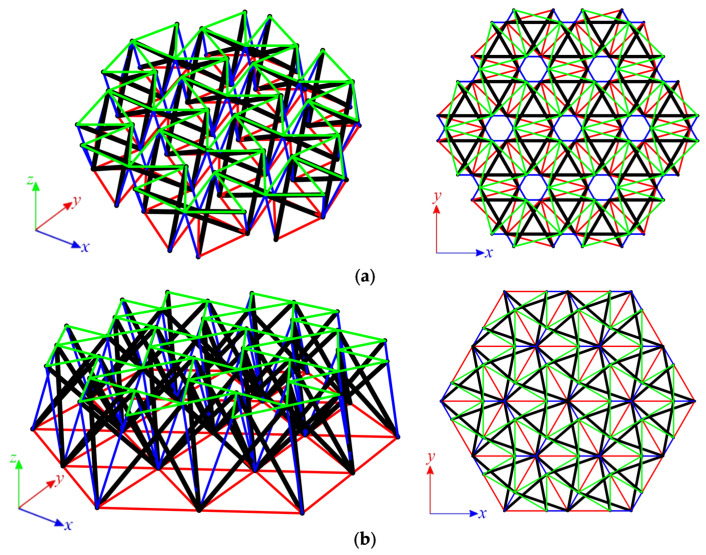
Six-module Simplex plate: (**a**) S24, (**b**) MS24.

**Figure 10:**
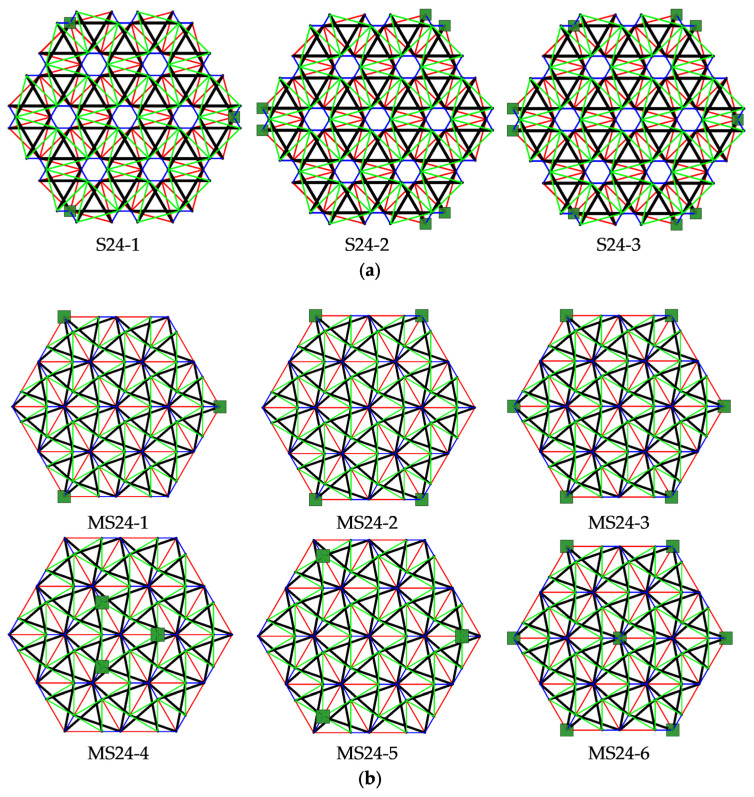
Scheme of support: (**a**) S24, (**b**) MS24.

As the last step, structures built with twenty-four Simplex modules are considered. The plate built with the S1 module (Figure 9a) consists of 288 elements (n = 288) and 84 nodes (w = 84), while the structures built with the MS1 module (Figure 9b) consist of 228 elements (n = 228) and 61 nodes (w = 61). Nine simply supported plates, with a different number of supported nodes, are taken into account (Figure 10).

For all considered twenty-four-module plates, the self-stress states are identified. Their number depends on the static scheme. Models S24-1, S24-2, and MS24-1 to MS24-4 are characterized by one mechanism. For these plates, similar to the six-module plates, self-stress states do not properly identify the type of elements, and the superimposed self-stress state is used for the identification of the type of the mechanism. The eigenvalues of the tangential stiffness matrix $K_{T1}$ are positive for all models with mechanisms; thus, the identified mechanisms are infinitesimal, and the structures are stable. With the presence of self-stress states, models S24-1, S24-2, and MS24-1 to MS24-4 are classified as structures with tensegrity features of class 1. The latter models, S24-3, MS24-5 and MS24-6, are qualified as structures with tensegrity features of class 2. The compiled results of the qualitative analysis of the twenty-four-module plates are presented in Table 4.

### 3.2. Quantitative Analysis

In the stage of the quantitative analysis, the influence of the level of initial prestress on the displacements **q**, the maximum effort $W_{\max}$ and the global stiffness parameter GSP is considered. The structures are assumed to be made of steel with Young modulus E=210 GPa and density $\rho =7860\;\mathrm{kg}/m^{3}$. The Halfen DETAN Rod System is adopted, and the following geometrical characteristics are assumed (the structures are built with the modules with bottom cables 1-m length; the parameter a presented in Figure 2 is a=1 m):

- For cables—made of round bars, steel S460N, diameter ϕ=20 mm.
- For struts—made of hot-finished circular hollow section, steel S355J2, diameter ϕ=76.1 mm, thickness t=2.9 mm.

The minimum prestress level depends on the support conditions and corresponds to the lowest level of prestress that ensures an appropriate identification of the type of elements, whereas the maximum is chosen to not cause the exceedance of the load-bearing capacity of elements.

#### 3.2.1. Single Simplex Module

**Figure 11:**
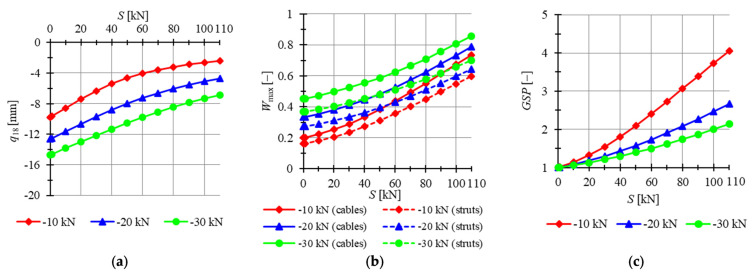
Impact of the initial prestress level *S* on the (**a**) maximum displacement $q_{z}$, (**b**) effort of structure $W_{\max}$, (**c**) global stiffness parameter GSP (model S1-1).

**Figure 12:**
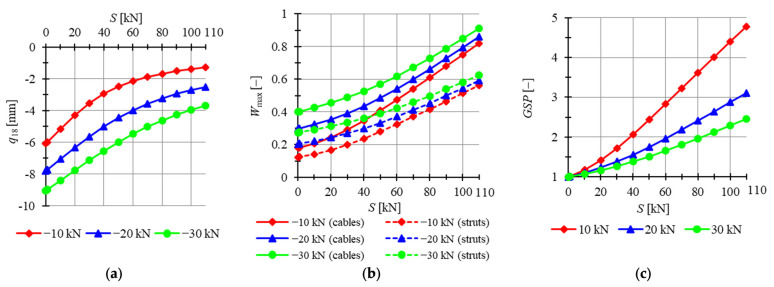
Impact of the initial prestress level *S* on the (**a**) maximum displacement $q_{z}$, (**b**) effort of structure $W_{\max}$, (**c**) global stiffness parameter GSP (model MS1-1).

The single Simplex modules are analyzed first. The ideal tensegrity modules are taken into account, so models S1-1 and MS1-1 are considered. For both modules, the minimum prestress level is $S_{\min}=0.01\;\mathrm{kN}$, whereas the maximum is assumed as $S_{\max}=110\;\mathrm{kN}$. A concentrated force applied vertically in the sixth node is considered. Three cases of the values of load are taken into consideration: $P_{18}^{1}=-10\;\mathrm{kN}$, $P_{18}^{2}=-20\;\mathrm{kN}$ and $P_{18}^{3}=-30\;\mathrm{kN}$. The influence of the level of initial prestress on the displacement $q_{18}$, the effort of structure $W_{\max}$ and the global stiffness parameter GSP are studied. For model S1-1, results are presented in Figure 11, and for MS1-1, in Figure 12.

The displacements obtained for the S1-1 model are on average 77.9% higher than for MS1-1. With the increase in the initial prestress, the displacements become lower for both models. For the minimal level of prestress, the difference between the displacements obtained for $P_{18}^{1}=-10\;\mathrm{kN}$ and $P_{18}^{2}=-20\;\mathrm{kN}$ is 20.5% for the S1-1 model and 20.8% for the MS1-1 model, while the difference between displacements for $P_{18}^{2}=-20\;\mathrm{kN}$ and $P_{18}^{2}=-30\;\mathrm{kN}$ are 12.6% and 12.8% for S1-1 and MS1-1 models, respectively. For the maximum level of prestress, the difference between the displacements calculated for $P_{18}^{1}$ and $P_{18}^{2}$ is 48.5% for S1-1 and 49.1% for MS1-1; the difference between displacements obtained for $P_{18}^{2}$ and $P_{18}^{3}$ is 30.3% for S1-1 and 31.5% for MS1-1. Comparing obtained values, it can be observed that with the increment in the initial prestress, the MS1-1 model becomes slightly more vulnerable to the increase in the load level. With the increase in the load, the impact of the level of prestress on the stiffness of the structure is more significant. More beneficial values of the GSP are obtained for the MS1-1 model. For that model, the increase in the level of initial prestress by 1 kN causes the increase in GSP by 0.0351, 0.0193 and 0.0132 for $P_{18}^{1}=-10\;\mathrm{kN}$, $P_{18}^{2}=-20\;\mathrm{kN}$ and $P_{18}^{3}=-30\;\mathrm{kN}$, respectively, while for S1-1 model, the increases are 0.0281, 0.0150 and 0.0107, respectively. The effort of cables is on average 22.6% higher than the effort of struts for the S1-1 model, while for MS1-1, it is 46.0%. The difference between the efforts increases with the increment in the prestress level and the rise in the load level. Generally, better results are obtained for the MS1-1 model.

#### 3.2.2. Six-Module Simplex Plate-like Structures

**Figure 13:**
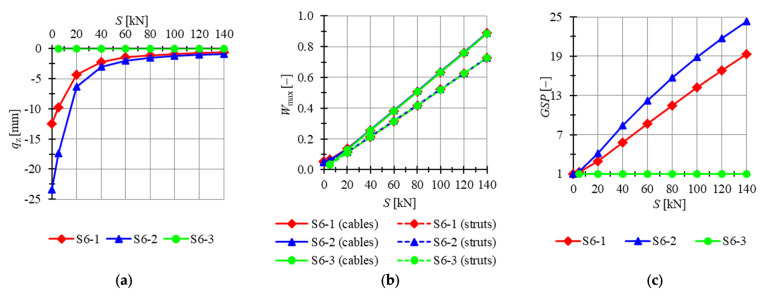
Impact of the initial prestress level *S* on the (**a**) maximum displacement $q_{z}$, (**b**) effort of structure $W_{\max}$, (**c**) global stiffness parameter GSP (six-module Simplex plate-like structures).

The six-module Simplex plate-like structures are considered in succession. Firstly, plates built with “normal” Simplex modules are analyzed. The minimum prestress level support is $S_{\min}=0.01\;\mathrm{kN}$ for S6-1 and S6-2 models and $S_{\min}=3\;\mathrm{kN}$ for the S6-3 model, whereas the maximum is assumed as $S_{\max}=140\;\mathrm{kN}$. Considered models are loaded by concentrated forces P=−1 kN applied to all top nodes of the structure. In Figure 13, the influence of the level of initial prestress on the maximum *z*-directional displacement $q_{z}$, the effort of structure $W_{\max}$ and the global stiffness parameter GSP is presented. For two out of three models (i.e., models S6-1, S6-2) mechanisms are identified, and for those structures, the initial prestress level affects the behavior of the structure. The last model, without mechanism (S6-3), is insensitive to the initial prestress level.

The lowest displacements are calculated for model S6-3. Regardless of the level of initial prestress, displacements are constants, and their values are close to zero. Displacements for model S6-2 are the highest; for the minimum level of initial prestress, the maximum displacement $q_{z}$ is 88.5% lower for the S6-1 model than for S6-2, while for the maximum level of initial prestress, the difference is lower and the displacement $q_{z}$ is 35.5% lower. The global stiffness parameter GSP is constant and equal to 1 for model S6-3. For models S6-1 and S6-2, the increase in the level of initial prestress by 1 kN causes the increase in the GSP by 0.1334 and 0.1729, respectively. The effort of cables is higher than the effort of struts by 13.1%, 12.1% and 2.14% for the minimum level of initial prestress and 21.8%, 21.8% and 22.0% for the maximum level of initial prestress for models S6-1, S6-2 and S6-3, respectively. The difference between the efforts of the structure is insignificant for the level of initial prestress higher than S=20 kN.

**Figure 14:**
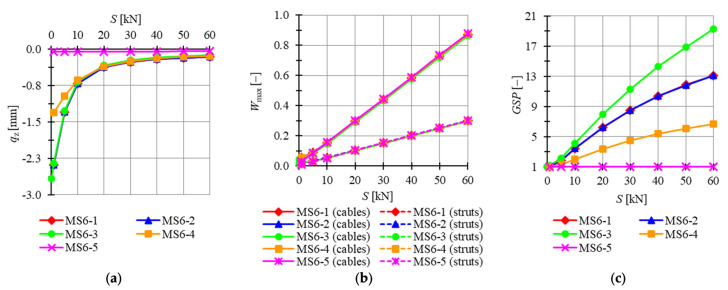
Impact of the initial prestress level *S* on the (**a**) maximum displacement $q_{z}$, (**b**) effort of structure $W_{\max}$, (**c**) global stiffness parameter GSP (six-module modified Simplex plate-like structures).

In succession, plates built with modified Simplex modules are taken into consideration. Four previously analyzed plates feature the mechanisms (i.e., models MS6-1 to MS6-4) and two are not (MS6-5 and MS6-6). For the latter analysis, modules MS6-1 to MS6-5 are chosen. The minimum prestress level is $S_{\min}=1\;\mathrm{kN}$ for MS6-1, MS6-2 and MS6-4 models and $S_{\min}=0.01\;\mathrm{kN}$ for the MS6-3 model, whereas the maximum is assumed as $S_{\max}=60\;\mathrm{kN}$. Considered models are loaded by concentrated forces P=−1 kN applied to all top nodes of the structure. In Figure 14, the influence of the level of initial prestress on the maximum *z*-directional displacement $q_{z}$, the effort of structure $W_{\max}$ and the global stiffness parameter GSP is presented.

For MS6-5, displacements are not correlated with the level of initial prestress and stay almost equal to zero for the whole range of the prestress levels. Comparing other models, the lowest displacements are obtained for MS6-4, and the highest for MS6-1. The difference becomes less significant with the increase in the level of initial prestress, and for the minimum level $S_{\min}$, the maximum z-directional displacement of model MS6-4 is 82.4% lower than for MS6-1 and 9.5% lower for the maximum level $S_{\max}$. Because of the lack of mechanisms in model MS6-5, the global stiffness parameter is constant and equal to 1. The increase in the level of initial prestress by 1 kN causes an increase in the GSP by 0.3169 for MS6-3; by 0.2113 for MS6-1 and MS6-2; and by 0.1009 for MS6-4. The effort of cables is higher than the effort of struts by 150.3%, 150.3%, 141.9%, 396.5% and 192.0% for the minimum level of initial prestress and by 188.7%, 188.7%, 188.8%, 192.0% and 192.0% for the maximum level of initial prestress for models MS6-1, MS6-2, MS6-3, MS6-4 and MS6-5, respectively. The difference between the efforts of the structure is insignificant for the level of initial prestress higher than S=5 kN.

#### 3.2.3. Twenty-Four-Module Simplex Plate-like Structures

**Figure 15:**
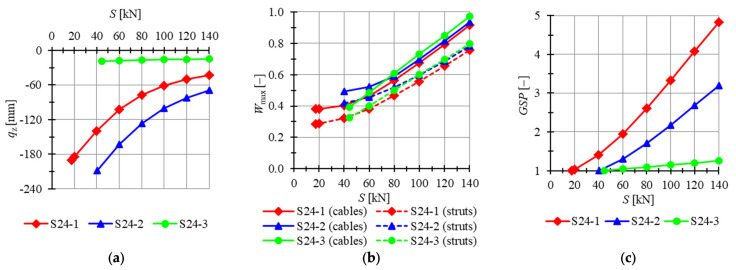
Impact of the initial prestress level *S* on the (**a**) maximum displacement $q_{z}$, (**b**) effort of structure $W_{\max}$, (**c**) global stiffness parameter GSP (twenty-four-module Simplex plate-like structures).

The twenty-four-module Simplex plate-like structures are taken into account next. Preliminarily, plates built with “normal” Simplex modules are analyzed. For two out of three models (i.e., models S24-1, S24-2), mechanisms are identified, and for the last model, S24-3, the level of the initial prestress state does not have any impact on the behavior of the structure. The minimum prestress levels for S24-1 to S24-3 are $S_{\min}=17\;\mathrm{kN}$, $S_{\min}=40\;\mathrm{kN}$ and $S_{\min}=44\;\mathrm{kN}$, respectively, whereas the maximum is assumed as $S_{\max}=140\;\mathrm{kN}$. Considered models are loaded by concentrated forces P=−1 kN applied to all top nodes of the structure. In Figure 15, the influence of the level of initial prestress on the maximum *z*-directional displacement $q_{z}$, the effort of structure $W_{\max}$ and the global stiffness parameter GSP is presented.

The lowest displacements are calculated for model S24-3. Displacements for model S24-2 are the highest; for the minimum comparable prestress level S=40 kN, the maximum displacement $q_{z}$ is 23.5% lower for S24-1 model than for S24-2 one, while for the maximum level of initial prestress, the difference is lower and the displacement $q_{z}$ is 30.8% lower. The increase in the level of initial prestress by 1 kN causes the increase in GSP by 0.0313, 0.0223 and 0.0026 for models S24-1 to S24-3, respectively. The GSP increases for the S24-3 model even though it is not characterized by the existence of mechanisms. The lowest maximum values of effort are obtained for model S24-1. For lower levels of prestress, the highest efforts are acquired for S24-2, but with the increase in prestress forces, S24-3 becomes less advantageous. The effort of cables is higher than the effort of struts for all models, and the difference between the efforts is not monotonically correlated with the level of initial prestress for models S24-1 and S24-2. For the S24-3 model, the differences in effort increase slightly with the level of initial prestress.

**Figure 16:**
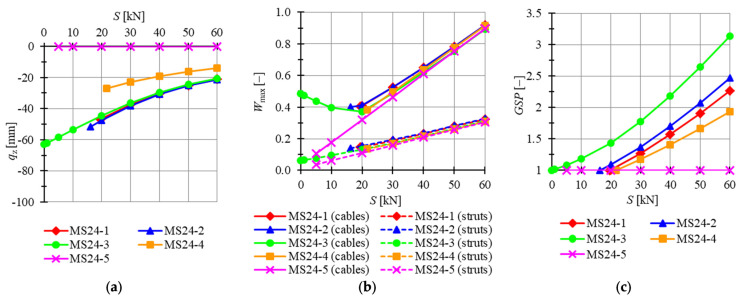
Impact of the initial prestress level *S* on the (**a**) maximum displacement $q_{z}$, (**b**) effort of structure $W_{\max}$, (**c**) global stiffness parameter GSP (twenty-four-module Simplex plate-like structures).

Consequently, plates built with modified Simplex modules are taken into consideration. Four previously analyzed plates feature mechanisms (i.e., models MS24-1 to MS24-4) and two do not (MS24-5 and MS24-6). According to the analysis of the six-module modified Simplex plates, the first five modules, MS24-1 to MS24-5, are chosen for consideration. The minimum prestress level is $S_{\min}=20\;\mathrm{kN}$ for MS24-1, $S_{\min}=16\;\mathrm{kN}$ for MS24-2, $S_{\min}=0.01\;\mathrm{kN}$ for MS34-3 and $S_{\min}=22\;\mathrm{kN}$ for MS6-4 and MS6-5, whereas the maximum is assumed as $S_{\max}=60\;\mathrm{kN}$. Considered models are loaded by concentrated forces P=−1 kN applied to all top nodes of the structure. In Figure 16, the influence of the level of initial prestress on the maximum *z*-directional displacement $q_{z}$, the effort of structure $W_{\max}$ and the global stiffness parameter GSP is presented.

For MS24-5, displacements are not correlated with the level of initial prestress and stay almost equal to zero for the whole range of prestress levels. Comparing other models, the lowest displacements are obtained for MS24-2 and the highest for MS24-4. The difference becomes less significant with the increase in the level of initial prestress, and for the lowest comparable level S=22 kN, the maximum z-directional displacement of model MS24-2 is 76.4% lower than for MS24-4 and 52.8% lower for the maximum level $S_{\max}$. Because of the lack of mechanisms in model MS6-5, the global stiffness parameter is constant and equal to 1. The increase in the level of initial prestress by 1 kN causes the increase in the GSP by 0.0335 for MS24-1, by 0.0311 for MS24-2, by 0.0347 for MS24-3 and by 0.0244 for MS24-4. The effort of cables is higher than the effort of struts by 175.4%, 189.0%, 679.2%, 185.1% and 192.0% for the minimum level of initial prestress and 182.8%, 179.2%, 187.0%, 196.8% and 192.0% for the maximum level of initial prestress for models MS6-1, MS6-2, MS6-3, MS6-4 and MS6-5, respectively. The difference between the efforts of the structure is insignificant for the level of initial prestress higher than S=20 kN.

## 4. Discussion

As numerous examples presented in this work show, the presence of the mechanisms depends on the geometry of the structure and on the support conditions. Taking into consideration different static schemes of the proposed structures, it can be noted that providing too many supports, the structures lose the existence of the mechanism. The displacements in the models without the mechanisms are lower compared to the rest of the models, but become invulnerable to the level of prestress. The effort of the structures stays on a similar level regardless of the support conditions, so the models with a limited number of supports, for example, due to environmental conditions, can exercise similar load-bearing capacity as their counterparts without the mechanisms. Comparing pairs of models, MS6-4/MS6-5 and MS24-4/MS24-5, it should be noted that not only the number of supports matters, but also their placements. Taking into account six- and twenty-four-module plates, for the same number of supports, the same number of mechanisms is obtained and only the number of self-stress states differs.

Generally, the more modules that are connected to multi-module structures, the higher the minimum level of self-stress becomes. Regardless of the number of clustered modules, the models have the same maximum level of self-stress $S_{max}$; for the models built with the “normal” Simplex module (S1), it is $S_{max}=60\;\mathrm{kN}$, while for the models built with the modified module (MS1), $S_{max}=140\;\mathrm{kN}$. For the single-module structures, the maximum level of self-stress is the same and $S_{max}=110\;\mathrm{kN}$. Comparing the single modules, more profitable behavior is obtained for the MS1 module. However, in the case of the multi-module structures, the examples built with the S1 module have better results, especially taking into account their load-bearing capacity.

Taking into account the global stiffness parameter (GSP), for the models characterized by the existence of mechanisms, the GSP increases almost linearly with the increment in the level of self-stress. The rise in the GSP is also related to the increase in the level of load. For the structures without mechanisms, GSP is constant and equal to 1, with the exception of S24-3, for which it increases slightly. For that example, relatively high displacements are obtained compared to the other models without mechanisms.

## 5. Conclusions

In this paper, we explored the possibility of the active control of stiffness of tensegrity plate-like structures throughout the change in the level of self-stress and the support conditions. We focused on the behavior of the structures built with the simplest tensegrity module, Simplex. Although in the literature, many structures without the necessary mechanisms are described as tensegrities, they behave significantly different from the structures featuring the infinitesimal mechanisms. For these structures, the control of static parameters is not possible, and only their shape resembles a tensegrity form. Therefore, the identification of the presence of the two most relevant tensegrity features, i.e., self-stress states and mechanisms, is a vital factor in order to properly classify and assess their behavior under external load. Numerous models considered in this paper confirm that the active control of tensegrity structures is possible; only then, the structure is classified as an ideal tensegrity or structure with tensegrity features of class 1, i.e., structure with mechanisms. For those classes, the stiffness of the structure, in addition to the geometry of the model and its material characteristics, also relies on the level of the prestress forces. The last class, the structures with tensegrity features of class 2, devoid of mechanisms, is insensitive to the change in the level of prestress.

This work focuses on the parametric analysis of the actual tensegrities, which are systems that can be controlled by the adjustment of the self-stress level. This area of research is still underdeveloped. The present research offers a better understanding of mechanical properties of tensegrity and shows that the tensegrity concept can be used not only as an avant-garde architectural design. The results presented in this paper confirm the feasibility of the active control of stiffness of tensegrity plate-like structures built with Simplex modules and gives a positive answer to the questions posed in the introduction. The behavior of the structure characterized by the presence of the infinitesimal mechanisms can be easily rectified by the change in the level of prestress and adjusted to the given conditions. This makes tensegrity a very promising structural concept, applicable in many areas when conventional solutions are insufficient, i.e., as temporary bridges and roofing of exhibition halls or sports stadiums. Future developments of this research can involve different deployment strategies that take into account various support conditions presented in this paper.

## Figures and Tables

**Table 1:** Numeration of elements in the single Simplex modules.

	Struts			Upper Cables			Bottom Cables			Middle Cables		
No. of element	1	2	3	4	5	6	7	8	9	10	11	12

**Table 2:** Results of the qualitative analysis of the structures built with the Simplex modules.

Model	No. of Nodes (w)	No. of Elements (n)	Degrees of Freedom (m)	No. of Self-Stress States	No. of Mechanisms	Classification
S1-1, S1-3, MS1-1, MS1-3	6	12	12	1	1	ideal tensegrity
S1-2, S1-4, MS1-2, MS1-4			9	4	1	structures with tensegrity features of class 1

**Table 3:** Results of the qualitative analysis of the six-module Simplex plates.

Model	No. of Nodes (w)	No. of Elements (n)	Degrees of Freedom (m)	No. of Self-Stress States	No. of Mechanisms	Classification
S6-1	24	74	63	10	1	structures with tensegrity features of class 1
S6-2			54	19	1	
S6-3			45	27	0	structures with tensegrity features of class 2
MS6-1	19	60	48	13	1	structures with tensegrity features of class 1
MS6-2			45	16	1	
MS6-3			39	22	1	
MS6-4			48	13	1	
MS6-5			48	12	0	structures with tensegrity features of class 2
MS6-6			36	24	0	

**Table 4:** Results of the qualitative analysis of the twenty-four-module Simplex plates.

Model	No. of Nodes (w)	No. of Elements (n)	Degrees of Freedom (m)	No. of Self-Stress States	No. of Mechanisms	Classification
S24-1	84	288	243	46	1	structures with tensegrity features of class 1
S24-2			234	55	1	
S24-3			225	63	0	structures with tensegrity features of class 2
MS24-1	61	228	174	55	1	structures with tensegrity features of class 1
MS24-2			171	58	1	
MS24-3			165	64	1	
MS24-4			174	55	1	
MS24-5			174	54	0	structures with tensegrity features of class 2
MS24-6			162	66	0	

## Data Availability

Data is contained within the article.
